# Raising the Level of Awareness of Nurse-to-Nurse Lateral Violence in a Critical Access Hospital

**DOI:** 10.1155/2013/207306

**Published:** 2013-08-06

**Authors:** Jennifer L. Embree, Deborah A. Bruner, Ann White

**Affiliations:** ^1^Indiana University School of Nursing, Indiana University-Purdue University, 1111 Middle Drive, NU W421, Indianapolis, IN 46202, USA; ^2^Minneola District Long Term Care and Minneola Clinics, Minneola District Hospital, 212 South Main Street, P.O. Box 127, Minneola, KS 67865-0127, USA; ^3^College of Nursing and Health Professions, University of Southern Indiana, 8600 University Boulevard, HP 2078, Evansville, IN 47712-3596, USA

## Abstract

*Background/Significance of Problem*. Nurse-to-nurse lateral violence (NNLV) has been internationally reported for greater than two decades and results in new nurse turnover and serious negative outcomes. *Clinical Question/Project Objective*. Will NNLV and cognitive rehearsal (CR) education result in a decrease in perceived nurse-to-nurse lateral violence in a critical access hospital (CAH)? The scope of this project was to determine perceived extent and increase awareness of NNLV through an educational project about NNLV and CR. *Clinical Appraisal of Literature/Best Evidence*. Trends of NNLV were assessed through an extensive literature review from Health Source, CINAHL, ProQuest Health, and Medical Complete. An educational forum about NNLV with CR was advocated for newly licensed nurses and current nurses (potential perpetrators of NNLV) with the goal of liberation of oppressed individuals. *Integration into Practice/Discussion of Results*. An interventional study with one group and pre-/postintervention was used to determine NNLV and CR education on perceived levels of lateral violence. Evidence-based measurement occurred through use of the Nurse Workplace Scale and the Silencing the Self-Work Scale. Outcomes were analyzed quantitatively through independent *t*-tests. Awareness of NNLV was increased. *Evaluation of Evidence-Based Practice/Implications*. Organizations must learn to eliminate NNLV. With increased levels of awareness of NNLV, nurses requested additional assistance in dealing with inappropriate behavior.

## 1. Introduction

Up to 90% of nurses experience lateral violence [1, 2]. Extensively and globally reported in the literature, nurse-to-nurse lateral violence (NNLV) or nurse aggression profoundly increases occupational stress with psychological, physical, and organizational consequences [3]. The pervasiveness of nursing workplace violence is of major concern for nursing as evidenced by the multiple position statements developed in response to NNLV [4]. A conservative estimate of the annual cost of nursing workplace violence is $4.3 billion dollars or nearly $250,000 per incident [5]. Nearly 60% of new nurses leave their initial employer within the first six months due to NNLV perpetrated in the workplace [6–8]. Each percentage point of nurse turnover results in an annual cost to an average hospital of nearly $300,000 and $3.6 million in poorly performing hospitals [9].

Utilizing effective evidence-based interventions can positively affect nurse-to-nurse interactions which in turn impact retention, recruitment, and a disenfranchised nursing work environment [10]. The substantive literature surrounding disruptive work environments elucidates the need for successful methods directed by nurse leaders to transform the organizational climate [11]. The current and projected nursing shortage of nearly one million nurses by 2020 can be reduced by evidence-based solutions to improve nurse-to-nurse interactions and enhance nursing retention [9]. Identifying nurse's perceptions of NNLV and integrating those insights into activities developed to halt NNLV afford nursing ownership of solutions. The purpose of this paper is to describe a nurse executive led study to determine the perceived extent of NNLV in a critical access hospital (CAH), to use a model to provide NNLV and cognitive rehearsal education as a shield for NNLV, and to break the cycle of nurse-to-nurse lateral violence.

Understanding the construct of lateral violence, its underlying determinants, and providing effective interventions are all key to stopping NNLV [12]. Nurse-to-nurse lateral violence occurs when oppressed groups or individuals internalize feelings such as anger and rage and display these emotions through behaviors such as gossiping, exhibiting jealousy, putting others down, and blaming others for their actions [13]. Nurses must recognize NNLV and the resulting negative consequences in order to change inappropriate behavior [12]. Within nursing, Griffin described lateral violence as “nurse aggression” [12]. Lateral violence (LV) is described as behavior demonstrated by nurses who overtly or covertly direct dissatisfaction toward those less powerful than themselves and each other [12]. Manifested verbally and nonverbally, the ten most common universal forms of LV in nursing are “nonverbal innuendo,” “verbal affront,” “undermining activities,” “withholding information,” “sabotage,” “infighting,” “scapegoating,” “backstabbing,” “failure to respect privacy,” and “broken confidences” [12].

A variety of terms for this disruptive behavior are found in the literature, and Griffin recommends naming the behavior as a first step to “calling out” the inappropriate behavior [12]. The author used the term nurse-to-nurse lateral violence for this project.

Interventions in the literature were reviewed prior to the selection of the lateral violence education and cognitive rehearsal technique used for the study. Scarcity of interventional studies addressing NNLV in the literature further suggested the need for effective techniques to deter NNLV [12]. De Marco found that nurses silence themselves as a strategy to avoid conflict and to maintain the status quo in the workplace and their private lives [14]. Griffin used cognitive rehearsal and lateral violence education to increase new nurses' awareness of this detrimental behavior and assist them in learning and practicing techniques that protected them from lateral violence [12].

Reviewed interventions included exploring NNLV behavior and its origins and praising and supporting each other [15–17]. Other interventions were managing lateral violence and intervention education and forming a writing group that developed a sense of support, connection, and voice [18]. Friere also identified the first step in altering a nurse's silence about lateral violence as understanding the cycle of the behavior and that awareness alone is often a liberating intervention [19]. Resources exist for nurse administrators to understand oppression and to utilize their understanding to break the cycle of NNLV. It is important to utilize these resources to improve disruptive behavior [20].

Developing mechanisms to educate nurses about oppressed group behaviors that result in NNLV can also enhance nurses' personal growth and development and improve their abilities to effectively combat the disruptive conduct [12]. One mechanism to decrease NNLV and improve nursing communication is a model of education to increase awareness of the behavior and to learn to use cognitive rehearsal [12]. Stagg and Sheridan identified that the best workplace bullying management programs in the literature included cognitive rehearsal of responses to inappropriate behaviors for staff nurses [21].

## 2. Methods

Nurses in critical access hospitals (CAHs) or hospitals with 25 or fewer inpatient beds are already challenged with limited resources due to their typical rural settings and less ability to select new personnel when nursing turnover occurs due to NNLV. Nurses in smaller organizations are at as much risk for NNLV as nurses in larger urban organizations. 

Initial interest in lateral violence education and cognitive rehearsal occurred after a presentation at a regional nurse executives' conference that featured Griffin's research [22]. Dr. Griffin's participants suggested using lateral violence and cognitive rehearsal education methodology with practicing RNs.

Expected outcomes of this project included decreased nurse perceptions of NNLV and reduced RN voluntary turnover. RN voluntary turnover in the year prior to project initiation was 7.84% with a national benchmark of 0.20% for hospitals under 100 beds [23]. Nurses represent the largest workforce in the project organization. Increased recognition of NNLV and development of cognitive rehearsal skills could enhance identification of inappropriate behaviors and provide support to dissuade the negative behavior among nurses in the organization [12]. 

The organizational economic profile included gross revenue of $66,000,000 and a net income of $1.5 million [24]. The organizational portfolio was a full service inpatient hospital housed in a CAH with 135 nurses at project onset. The chief clinical officer (CCO/nurse executive) led and supported the project in conjunction with the chief executive officer and the organization. The project was approved by a university institutional review board and the hospital medical executive committee since the organization did not have an institutional review board.

When undertaking an interventional project, it is important to identify necessary internal marketing needs [25]. The marketing plan for this endeavor included defining the project, competition (for staff time), and forecasting environmental change [25]. Pull strategy was used to introduce the project with the belief that nurses would request the education from the project director if they did not attend the initial focus groups [25]. Development of a strategic mindset focused on the customer (nurse) [25]. Focusing on the nurse was based on the concept of serving a particular target (nurses) in an exceptional manner, because of minimal competition (time) and high profit margins (project success/improved work environment) [25]. If nurses maintained their current position in the organization and the hospital experienced a decrease in turnover and vacancy costs related to the intervention, the resultant expense reduction could improve the profit margin by offsetting expenses [25].

Eligible nurses for the study included nurses employed in roles in physician offices, hospital staff, nursing leadership, and other nonpatient care roles. Recruitment efforts included repeated personal, face-to-face invitations, flyers, e-mails, and notification through nursing leaders. Nurses were paid by the organization for the time they participated in activities associated with the project. 

## 3. Instruments

Two instruments were used to measure behaviors, beliefs, or feelings which the nurses perceived that they experienced. These behaviors are related to NNLV. The instruments would also detect if there were a change in perceptions and responses after learning about origins, attributes, and interventions for NNLV. These measurement tools were the Nurse Workplace Behavior Scale (NWS) and the Silencing the Self-Work Scale (STSS-W).

 “Internalized sexism” and “minimization of self” reflecting oppressed group behaviors, beliefs, or feelings the nurse experienced were measured using the NWS [25]. The NWS questions ask how often the respondent has engaged in outcome measures congruent with nurse empowerment, and a higher score equals greater perceptions of empowerment. These concepts identified nurses' ability to adapt or fail to acclimate to their work environments [25]. 

The NWS is a reliable measure of lateral violence (LV) and professional esteem. Testing of this instrument determined that oppressive group behaviors exist in nurses, varying by age and practice area. Ability to measure lateral violence and self-esteem has great potential for development of research and practice strategies to improve nursing interactions [26].

The STSS-W measures behaviors congruent with self-silencing. De Marco also found that nurses silence themselves as a strategy to avoid conflict and to maintain the status quo in the workplace and their private lives [14, 27, 28]. De Marco's study utilized the STSS-W for nurses, based on the original “Silencing the Self-Scale” of Jack [29, 30]. The scale has subsequently been tested with a random sample of nurses and was found to be a reliable and valid instrument with nursing samples [28]. The instrument is a useful tool to document silencing in nursing and to evaluate interventions aimed at change. The NWS and the STSS-W were utilized for the study after review of the literature and communication with Griffin, the investigator who initially utilized cognitive rehearsal in her work with newly graduated nurses [31]. 

## 4. Validity and Reliability

Permission for usage of the NWS and the STSS-W was provided by De Marco [32]. The NWS validity and reliability analyses were acceptable: the oppressed self-factor and oppressed group factor Cronbach's alpha were .81 and .78. The full 12-item scale was .81 Cronbach's alpha [26]. The NWS was arranged in a Likert-style format so that rating for agreement ranged from never to consistently, on a 1 to 5 scale. The questions asked how often a respondent engaged in specific activities, feelings, or beliefs which were examples of oppressed group behavior in nursing practice [26]. 

Examples of behaviors measured in the NWS are (a) “gotten more frequently regarded by others (not patients) for the performance of technological tasks than for meeting the psychological needs of patients;” (b) “complained about a problem to your fellow nurses but did nothing to confront the person you believe is causing the problem;” and (c) “felt that nurses in power positions ‘over you' have more loyalty to physicians/administrators than they do to nursing” [26].

The STSS-W analysis of validity and reliability exhibited alpha scores of internal consistency ranging from .86 to .94 on the total STSS-W scores. Test-retest reliability ranged from .88 to .93. The STSS-W represents behaviors based on judging oneself externally, putting others' needs first, refraining from self-expression, conflict-avoidance, and relationship loss [28].

The NWS measures a feeling or belief that is an example of an oppressed individual or group behavior in nursing practice. The NWS has two components “internalized sexism” (5–25) and “minimization of self” (7–35) behaviors. The total NWS scale score is 12–60 and represents oppressed group behaviors, beliefs, or feelings [26].

Evidence supports using education and cognitive rehearsal to raise the level of awareness of NNLV and shield the negative effects of the disruptive behavior on learning and socialization [12]. In order to adequately assess the extent of NNLV, all nurses at a CAH (*n* = 135) were surveyed to determine perceptions of NNLV in the organization. The pre-survey included demographics as well as the NWS and the STSS-W. Upon completion of the pre-survey, the author developed an educational and cognitive rehearsal intervention. 

The intervention included didactic content of theoretical underpinnings and historical significance of NNLV and cognitive rehearsal. Handouts for dealing with confrontation and conflict, cue cards of NNLV responses, and expected behaviors of professionals supplemented the didactic education. Teaching strategies aimed to increase knowledge, self-esteem, and comfort with information and techniques. Two-hour focus sessions were provided to interested nurses. Three sessions at a variety of times were offered with only eight nurses attending. Additional sessions were offered without additional staff participation. Recruitment efforts as previously identified included repeated invitations through a variety of venues with minimal participants engaged. 

The project director/nurse executive conducted the intervention within the hospital for nursing staff convenience. During the cognitive rehearsal session, nurses identified personal experiences with NNLV and offered suggestions to other participants about mechanisms they had found successful in handling NNLV. Nurses agreed to report future episodes and responses of NNLV to the project director/nurse executive over the project time-span and to discuss management of those responses to improve their skills in dealing with NNLV within the organization. See curriculum, Table 1.

Six to nine months after education, all nurses (*n* = 143) were surveyed to determine their perception of the extent of NNLV among nurses in the organization. Previous focus education participants were invited to attend posteducation focus groups to discuss additional management strategies for NNLV. Due to three hospital realignment strategies during the course of this three-year project, not all participants were asked to complete the postfocus groups. The five staff invited to participate determined that online survey was their strategy of choice for completion of their project involvement, and all five nurses completed the survey. Results of the postparticipation online survey as well as focus group dialogue are outside the scope of this paper. 

## 5. Measure of Nurse-to-Nurse Lateral Violence

### 5.1. Results

Thirty-five percent (48) of the 135 CAH nurses participated in the pre-survey. Twenty-four percent (35) of the 143 nurses participated in the post-survey. Focus group participants totaled eight nurses for the pre-survey. Survey items with missing data were excluded from analysis. 

Participant age ranges were requested to preclude participant responses being identified in this smaller organization. Forty-two percent of pre-survey nurses were 35–45 years old. Thirty-nine percent of post-survey nurses were 45–55 years old. Fifty percent of focus group participants were 35–45 years old. Seventy percent pre-survey and fifty-eight percent post-survey respondents identified that they worked in staff roles.

Fifteen percent of participants were nationally certified (Table 2). Eighty-two percent pre-survey and sixty-seven percent post-survey nurses identified a religious or spiritual affiliation. All focus group participants reported religious or spiritual affiliation. The majority of study participants were RNs. The six percent change in LPN participants from pre- to post-survey may have been related to internal organizational changes that resulted in role changes.

Forty-two percent of pre-survey nurses worked in critical care areas (Table 3). The nurse executive was the former critical care manager, and this may have impacted the critical care nurses participation. No post-survey respondent trends were noted. The majority of respondents for all surveys worked full time day shift positions.

### 5.2. Measurement Results

#### 5.2.1. Internalized Sexism Results

The Internalized Sexism Scale measures behaviors which minimize the effectiveness of actions (Table 4). Possible ranges for Internalized Sexism were 5 to 25. Results of 8.95 in the pre-survey and 8.66 in the post-survey demonstrate downward trending of means for the NWS-Internal Sexism Scale. The downward trending could indicate that respondents were demonstrating less personal oppression due to increased awareness of NNLV and feelings of improved empowerment and self-esteem [26]. 

#### 5.2.2. Minimization of Self

Possible ranges of the Minimization of Self or “oppressed group” were 7 to 35. Results of the oppressed group scale of 19.02 for the pre-survey and 20.06 for the post-survey indicate a slight upward trend in means. The increase in the Minimization of Self may be related to the perception of less oppressed group movement towards liberation rather than personal oppression (Internalized Sexism Scale). Nurses may feel enhanced personal empowerment but less group empowerment (Table 4).

#### 5.2.3. Total NWS Survey

The total NWS post-survey demonstrated a slight increase in means, which could indicate that while NNLV recognition may be occurring and the “oppressed self” has improved empowerment, the “oppressed group” has not made movement, and some minimization of self is still occurring, as previously discussed (Table 4). Nursing staff may still be demonstrating less personal oppression or have improved their coping mechanisms for NNLV. Nurse respondents also may be adapting and responding appropriately to NNLV on a personal level but still not speaking out against NNLV. Possible ranges for the NWS Total Scale are 12 to 60. The total NWS had an increasing mean from 27.97 pre-survey to 28.72 post-survey. The NWS provides a conceptual match of behaviors that limit the effects which nurses have as leaders and change agents [26].

The STSS-W measures the ability to express personal needs or feelings directly or put others first. The STSS-W range is 25 (low) to 125 (high). Scoring higher on the STSS-W may mean that the organization reinforces these behaviors [28]. Decreasing means from 67.02 pre-survey to 65.18 post-survey may suggest that nurses were beginning to rely less on external judgment, were putting themselves first, and were feeling more empowered to speak up than to silence themselves. Results of the project included trending means (a decrease) for the NWS-Internal Sexism and the STSS-W (Table 4).

RN voluntary turnover is impacted by multiple variables. Voluntary turnover was defined as the total number of full-time and part-time RNs and advanced practice nurses voluntarily separating from an organization during a calendar year (Table 5). Voluntary turnover was 7.84% in the study organization the year prior to the study initiation [23]. The benchmark was the mean turnover for hospitals less than 100 beds from the national database of nursing quality indicators [23]. Voluntary nursing turnover in the study organization hovered around 7% from 2002 until 2008 [23]. During this study the hospital was in merger discussions and experiencing reimbursement issues related to healthcare instability, so any changes in turnover must be viewed cautiously.

## 6. Discussion

Data analysis for pre- and post-survey data identified no statistical significance. However, when reviewing trends, the means of the NWS-Internal Sexism Scale and the STSS-W indicate a positive sense of empowerment and self-esteem [26, 28]. The survey trending results were further supported in anecdotal data during the time of the study (personal communication). Nursing staff identified expression of discomfort with NNLV and addressed potentially contentious issues that may adversely affect the work environment [33].

## 7. Limitations

While trends were identified, results of the study should be viewed with caution. The study occurred in one CAH hospital, so generalizability of the small sample and of this study cannot be expanded to other CAHs or other healthcare facilities. In addition, the project director was the chief clinical officer/nurse executive in the CAH. As the leader, gentle encouragement for participation could be utilized, but any pressure to participate would be unacceptable. 

Initial project education with leadership at all levels is crucial. Several roles in the facility were realigned during the project due to change in accountability for specific service areas related to behaviors, better fit, and organizational need. This methodology was previously untested with registered nurses. Complex confounding variables include organizational realignment, acquisition by a major faith-based organization, and continued financial challenges related to healthcare reform.

## 8. Conclusions

Nursing must be compelled to reduce nurse-to-nurse lateral violence [18]. Nurse executives must be exemplars for affirmative culture change in healthcare facilities. As organizations are continually challenged to improve quality, decrease expenses, improve efficiencies, and enhance nursing excellence, it becomes increasingly important to improve nurse-to-nurse interactions [34]. The project provided nursing dialogue for contentious situations in a CAH. Post-survey participants described recognizing their personal displays of NNLV and intervened when witnessing lateral violence in other nurses [33].

When implementing a study in an organization where participation is optional, marketing is critical to project success from gaining initial stakeholder support to garnering participants in the endeavor [25]. One must assume that recruitment is complex, especially with uncomfortable topics. Staff is busy, and they choose how they will spend their time. Greater study participation may have occurred in another organization where the author was not the nurse executive. Flexibility regarding project execution was critical to successful implementation and progression. Study timelines were adjusted due to organizational demands and changes in reporting structures related to hospital acquisition.

Using the intervention of NNLV education and cognitive rehearsal as a response to NNLV in the small group did not appear to encourage usage of cognitive rehearsal but increased awareness of NNLV. As Griffin found, it was unclear whether cognitive rehearsal or just raising consciousness about NNLV and learning and practicing appropriate responses were helpful in this small focus group [12]. In the organization, nurse-to-nurse lateral violence education was added to annual nursing education. Multiple regional, state, national, and international organizations have requested presentations to their nurses about NNLV and cognitive rehearsal after initial dissemination and discussion of this project occurred at a university nursing research conference.

Monitoring the work environment for signs of ineffective communication and NNLV is critical to emphasize the importance of exhibiting appropriate behavior [12]. The project director annually evaluated the lateral violence education program and adjusted content and interventions based on feedback. Nurses were encouraged to share stories of NNLV with other nurses to assist in decreasing the problem behavior by describing effective methods of deflecting this behavior.

This project could be piloted in other critical access hospitals controlling for variables that confounded this project. Extending interventional research directed at development of interpersonal and organizational strategies for preventing and addressing NNLV could assist in improving poor work climates [12].

Education about NNLV could be expanded to other organizations and to nursing school curriculums. The potential increased level of awareness and enhanced skill may assist in decreasing the number of new nurses leaving their first role [12]. Clarification is needed about the effect teaching and learning about NNLV have on diminishing the disruptive behavior [12]. To combat the future nursing shortage, it is essential to retain newly registered nurses and to recruit additional nurses into the workforce.

When an organization possesses a reputation of harboring a negative practice environment by allowing NNLV, recruitment efforts are difficult and retention is problematic [14]. The projected RN shortage and pervasiveness of NNLV serve as exemplification of need for interventions to deter NNLV and retain current RNs in the workforce [9]. Organizations must increase the level of awareness of NNLV and hold all accountable for their behavior [12].

## Figures and Tables

**Table 1:** Nurse-to-nurse lateral violence education and cognitive rehearsal curriculum.

NNLV education	Cognitive rehearsal education
Objectives	Cognitive process
Best evidence	Prevention intervention
Concept map	Cognition
Professional behaviors	Cognitive learning theories
Healthcare outcomes	Cognitive rehearsal
Practice impact	Interventions
System change	Plan for education
	Role play

**Table 2:** Educational preparation.

Survey	LPN	RN	ASN	BSN	MSN	Masters not in nursing	APN
Pre-survey (*n* = 44)	9%	85%	51%	36%	6%	0%	6%
Post-survey (*n* = 33)	3%	91%	39%	42%	3%	6%	6%
Focus group (*n* = 8)	24%	87%	24%	50%	0%	0%	0%

**Table 3:** Nursing work areas.

Survey timeframe	Medical surgical	Critical care	Obstetrics	Surgical services	Emergency services	Outpatient clinics/MD offices	House wide	Homecare
Pre-survey (*n* = 44)	24%	42%	15%	3%	6%	6%	12%	3%
Post-survey (*n* = 33)	15%	27%	9%	18%	15%	12%	3%	3%
Focus group (*n* = 8)	25%	12%	12%	0%	0%	0%	50%	0%

**Table 4:** Internalized Sexism, Minimization of Self, Total NWS, and STSS-W.

Survey timeframe	Survey	Survey	Survey	Survey
	Internalized Sexism	Minimization of Self	Total NWS	STSS-W
Pre-survey	8.95	19.02	27.98	67.03
Post-survey (1 year later)	8.67	20.06	28.73	65.19

**Table 5:** RN voluntary turnover.

RN turnover	Before year 1	Year 2	Year 3
Benchmark^a^	0.20%	0.15%	0.20%
Hospital^a^	7.84%	1.42%	0%

^a^NDNQI Hospital Data 2008, 2009, and 2010 [23].
